# The association between educational level and dementia in rural Tanzania

**DOI:** 10.1590/S1980-57642014DN82000006

**Published:** 2014

**Authors:** Stella-Maria Paddick, Anna Longdon, William K. Gray, Catherine Dotchin, Aloyce Kisoli, Paul Chaote, Richard Walker

**Affiliations:** 1 Institute of Ageing and Health, Newcastle University, Newcastle upon Tyne, UK.; 2 South Devon Healthcare Trust, Exeter, UK.; 3 Northumbria Healthcare NHS Foundation Trust, North Tyneside General Hospital, North Shields, UK.; 4 Hai District Hospital, Boma’ngombe, Kilimanjaro, Tanzania.; 5 Institute of Health and Society, Newcastle University, Newcastle upon Tyne, UK.

**Keywords:** dementia, education, schooling, Tanzania, Africa

## Abstract

The majority of people with dementia worldwide live in developing countries. Studies from the developed world have reported an association between lower educational attainment and dementia, but there are few data from the developing world where literacy and educational levels are frequently much lower. In this study we assessed the association between education and dementia prevalence in a rural Tanzanian setting.

**Methods:**

In phase I, 1198 individuals aged 70 and over were assessed using the Community Screening Instrument for Dementia (CSI-D). In phase Ii a stratified sample of those seen in phase I were fully assessed and a clinical diagnosis based on DSM-IV criteria was made where appropriate. Information regarding literacy, highest attained educational level and occupation were also collected.

**Results:**

The median subject cognitive score on the CSI-D was 25.7 (IQR 22.7 to 28.0) for females and 27.7 (IQR 25.7 to 29.4) for males. This difference was significant (U=117770.0, z= –9.880, p<0.001). In both males and females a lower CSI-D subject cognitive score was significantly associated with having had no formal education (U=34866.5, z= –6.688, p<0.001, for females; U=20757.0, z= –6.278, p<0.001, for males). After adjusting for the effect of age, having no formal education was significantly associated with greater odds of having 'probable dementia' by CSI-D, as was illiteracy. Amongst those interviewed in phase II, there was no significant difference in literacy or education between those with diagnosed DSM-IV dementia and those without.

**Conclusion:**

In this rural Tanzanian population, we found a significant association between low levels of education and dementia by CSI-D. This relationship was not significant in cases meeting DSM-IV criteria for dementia.

## Abstract (pt)

A maioria das pessoas com demência no mundo vivem em países em desenvolvimento. Estudos realizados em países desenvolvidos têm relatado uma associação entre baixa escolaridade e demência, onde os níveis de alfabetização e educação são frequentemente muito mais baixos. Neste estudo avaliou-se a associação entre a educação e a prevalência de demência em um cenário rural da Tanzânia.

**Métodos:**

Na fase I, 1.198 indivíduos com 70 anos ou mais foram avaliados utilizando o Instrumento de Rastreamento Comunitário para Demência (CSI-D). Na Fase II uma amostra estratificada dos pacientes avaliados na fase I foram totalmente avaliados e um diagnóstico clínico baseado em critérios do DSM-IV foi feito quando necessário.

**Resultados:**

A mediana do escore cognitivo no CSI-D foi de 25,7 (IQR 22,7-28,0) para o sexo feminino e 27,7 (IQR 25,7-29,4) para o sexo masculino. Esta diferença foi significativa (U=117770,0, z= –9,880, p <0,001). Em ambos os sexos, masculino e feminino a pontuação cognitiva menor no CSI-D foi significativamente associada com ausência de educação formal (U=34866,5, z= –6,688, p <0,001, para as mulheres; U=20757,0, z= –6,278, p<0.001, para o sexo masculino). Após o ajuste para o efeito da idade, ausência de educação formal foi significativamente associada com maiores chances de ter provável demência pelo CSI-D, como foi para o analfabetismo. Entre os entrevistados na fase II, não houve diferença significativa na alfabetização ou de educação entre aqueles com ou sem diagnóstico de demência do DSM-IV .

**Conclusão:**

Nesta população da Tanzânia rural, encontramos uma significativa associação entre baixos níveis de educação e demência pelo CSI-D. Esta relação não foi significativa em casos que preencheram os critérios do DSM-IV para demência.

## INTRODUCTION

Dementia is becoming a worldwide public health issue, with 44.35 million people currently estimated to have the condition.^1^ The resultant disease burden is considerable in terms of disability and health resource utilisation. Since curative treatment is not currently available, preventive strategies and identification of potentially modifiable risk factors are the priority. Strategies to improve quality of life for people with dementia and their carers are also urgently needed.

In sub-Saharan Africa (SSA), it is estimated that there are currently 2.1 million people with dementia.^2^ Recent data suggest that prevalence of dementia in the elderly is currently similar to that reported in high income countries^3-6^ and will continue to rise as demographic transition continues. In fact, 71% of people with dementia will live in low and middle income countries (LMIC) by 2050.1 Identification of potentially modifiable risk factors relevant to these settings is therefore even more pressing.

Cognitive reserve is a potentially modifiable risk factor which is currently the subject of much research interest.^7-10^ The concept originates from evidence that there is often little relationship between observed degree of neuropathology and cognitive impairment, with some individuals able to tolerate a much greater degree of disease burden without demonstrating clinical evidence of dementia.^7,11,12^ Educational exposure is thought to increase cognitive reserve through neuroplasticity and creation of more complex neural networks, resulting in the ability to compensate for greater degrees of neuropathology in later life.^11^ Education is therefore frequently used as a proxy for cognitive reserve in studies.^13^

In high-income countries, educational attainment is consistently associated with reduced dementia risk^14-17^ as well as a delay in onset of the dementia syndrome. Meta-analyses report relative risk (RR) of 1.59 for all dementias for those with lower education and pooled OR of 2.61 (95% CI 2.21-3.07).^13-18^ The association appears greater for Alzheimer's disease (AD) with a RR of 1.88.18The majority of these studies include subjects with relatively high levels of education in high-income countries. It follows that they cannot be generalised to low-income countries where significant numbers of elderly people are illiterate. Nevertheless, illiteracy appears to also have a consistent association with dementia.^19^

In LMICs, data on education and dementia risk are few and the association appears less consistent.^20^ In general, the relationship between dementia and education level appears to be weaker in settings where access to education is lower. Studies from Korea, China,^21-23^ Egypt^24^ and Arab populations in Israel^25^ have reported illiteracy or less than one year of school attendance to be associated with dementia, whereas studies from India^26,27^ did not find a significant association. These studies report overall illiteracy or 'no education' rates of between 11%^27^ and 73%.^26^

In SSA, data on educational level and dementia is even more limited. Of the small number of studies which have been published, the majority report no significant relationship between educational level and dementia,^28-32^ with the exception of one study.^33^ The overall educational level reported in dementia prevalence studies from SSA is also low, with quoted illiteracy rates of 47%^31^ to 96.6%.^4^

In low literacy settings the situation is further complicated by difficulties in assessing cognition with the possibility of false positives on dementia screening if assessment tools designed in high-income settings with higher educational levels are used.^34,35^

Low education may also be a marker of general social disadvantage and may be difficult to separate from other socioeconomic factors associated with dementia risk such as rural residence. In settings where access to education is low across the population, other markers of cognitive reserve have been suggested. These include occupational attainment and self-reported literacy as these may reflect learning outside formal educational settings.

The aim of this study was therefore to assess the relationship between measures of cognitive reserve including self-reported education, literacy, occupational level and dementia risk in a rural Tanzanian population of similar socio-economic background using cognitive assessment tools designed for low-literacy settings.

## METHODS

These data were collected as part of a two phase community-based dementia prevalence study in the Hai district, Northern Tanzania. This study and full methods have been published elsewhere,^3^ so only a brief summary will be presented here.

**Population.** The Hai district is a rural area on the slopes of Mount Kilimanjaro. The majority of the population are subsistence farmers, but some families are able to produce cash crops such as coffee and tomatoes. The majority of the population are of Chagga origin, with a smaller proportion in the lowland areas being Maasai. The Hai district is a demographic surveillance site (DSS) and therefore regular censuses are conducted. Using census data, trained village health workers invited all persons aged 70 and over residing in six representative villages to take part in the study.

**Assessments.** All participants were screened for dementia in phase one using the Community Screening Instrument for Dementia (CSI-D)^36^ by local village health workers who had been trained in its use by members of our team. This screening tool was specifically designed for use in developing countries and in low literacy settings and includes an informant interview. The CSI-D has been used extensively in developing countries more recently as part of the 10/66 dementia research group protocol^35^ and has been validated in Swahili in neighbouring Kenya.^37^ The CSI-D has two parts; a patient cognitive screen (COGSCORE) in which the subject is asked a series of questions and asked to complete cognitive tasks and a second section completed by a close relative or friend acting as an informant, which includes questions related to functional performance, such as activities of daily living (RELSCORE). The scores for the two sections can be completed to give an overall score that can be used to rank individuals into 'probable dementia', 'possible dementia' and 'no dementia' categories.

All participants were asked specifically about educational attainment in terms of grade reached in school and whether they had ever learned to read or write. Literacy was specifically asked about separately to take into account the fact that some participants might have been taught informally by family members despite never having attended formal school. All participants were asked to give primary and secondary occupation, if applicable, in view of the fact that most people worked in agriculture even if they had another source of income.

In phase II, we aimed to fully assess all people with 'probable dementia', at least 50% of people with 'possible dementia' and at least 5% of people with 'no dementia'. During clinical assessments subjects were interviewed by a research doctor using the 10/66 Dementia Research Group protocol.^35^ This includes the Geriatric Mental State (GMS),^38^ a neurological examination, a detailed background risk factor interview and the neuropsychiatric inventory (NPI).^39^ The Consortium for Research into Alzheimer's Disease (CERAD) 10 word learning list^40^ was also administered as part of the protocol. This task has been extensively validated across developing countries and scores appear consistent across cultures. A diagnosis of dementia was made according to the DSM-IV criteria^41^ using all information available. Diagnoses were subsequently checked by a UK-based specialist in old age psychiatry.

**Ethics.** This study was approved by the National Institute of Medical Research, Dar-es-Salaam, Tanzania and by the Newcastle and North Tyneside Joint Ethics Committee in the UK. Signed informed consent was obtained from each participant. We obtained a thumbprint for those that could not read and write. The purpose and implications of the study were verbally explained. In cases where patients were unable to give valid consent, written assent was obtained from a close relative.

**Statistics.** Data were analysed using standard statistical software, PASW-18 for windows (PASW, Chicago, IL, USA). All data were non-normally distributed and so non-parametric tests were used. Data are described in terms of the median and inter-quartile range (IQR). Mann-Whitney U test (ordinal, interval and ratio data) and odds ratios (categorical data) were used to test differences between groups. Logistic regression modelling was used to investigate the influence of multiple variables on key outcomes. The significance level was set at 5% and two-tailed tests were used throughout.

## RESULTS

**Phase I screening.** The six villages had a population of 1,260 people aged 70 and older on the prevalence date, of whom 1,198 people were screened. Of those screened, 673 (56.2%) were female. From CSI-D screening in phase I, 184 people (15.4%) had 'probable dementia', of whom 125 (67.9%) were female. A further 104 people (8.7%) had 'possible dementia', of whom 68 (65.4%) were female. The remaining 910 (480 female, 52.7%), were classified as 'no dementia'. The median subject cognitive score on the CSI-D was 25.7 (IQR 22.7 to 28.0) for females and 27.7 (IQR 25.7 to 29.4) for males. This difference was significant (U=117770.0, z= –9.880, p<0.001). Educational data were available for 1,186 people (99.0%). Overall educational level was remarkably low. Of 668 females, only 44 (6.8%) had more than 4 years of education, 205 (30.7%) had 4 years or less, with 419 (62.7%) having had no education at all. Educational level was generally higher in males with 96 (18.5%) reporting more than 4 years of education, 256 (49.4%) had 4 years of education or less and 166 (32.0%) having had no education at all. Females were 3.56 times (95% CI 2.80-4.55) more likely to have had no education than males. A small number of participants had received education through adult literacy classes. Data on literacy (being able to read and write) was also available for 1186 subjects. Place of birth (Hai district or outside Hai) and ever having lived outside Hai district were available for all subjects. Median CSI-D COGSCORES for each of these variables by gender are presented in Table 1. The association between literacy and schooling was strong. Only 12 subjects (1.0%) with no formal education were able to read and write suggesting that informal education was uncommon in this setting. Of those who had attended school, only 44 subjects (3.7%) were unable to read and write. Both schooling and literacy were significantly associated with higher CSI-D cognitive scores in both males and females.

**Table 1:** Literacy, schooling and life experience data in relation to CSI-D cognitive score

		Yes			No		Significance
		Number	CSI-D score (median, IQR)		Number	CSI-D score (median, IQR)	
Females	Ever attended school (n=668)	249 (37.3%)	26.9 (24.7 to 28.9)		419 (62.7%)	24.8 (21.6 to 27.4)	U=34866.5,z= -6.688, p<0.001
	Can read and write (n=668)	232 (34.7%)	26.9 (24.7 to 28.7)		436 (65.3%)	24.9 (21.8 to 27.5)	U=34449.5, z= -6.791, p<0.001
	Born outside Hai (n=673)	70 (10.4%)	25.2 (23.6 to 27.3)		603 (89.6%)	25.8 (22.6 to 28.1)	U=19397.0, z= -1.109, p=0.267
	Ever lived outside Hai (n=673)	96 (14.3%)	26.1 (24.1 to 28.0)		577 (85.7%)	25.6 (22.6 to 28.0)	U=26581.0, z= -0.632, p=0.527
Males	Ever attended school (n=518)	352 (68.0%)	28.3 (26.3 to 29.9)		166 (32.0%)	26.5 (24.0 to 28.0)	U=20757.0, z= -6.278, p<0.001
	Can read and write (n=518)	337 (65.1%)	28.2 (26.3 to 29.8)		181 (34.9%)	26.8 (24.0 to 28.6)	U=21481.5, z= -6.039, p<0.001
	Born outside Hai (n=525)	66 (12.6%)	27.1 (26.1 to 28.3)		459 (87.4%)	27.9 (25.5 to 29.5)	U=13771.5, z= -1.194, p=0.233
	Ever lived outside Hai (n=525)	157 (29.9%)	28.0 (26.2 to 29.6)		368 (70.1%)	27.5 (25.3 to 29.4)	U=26422.5, z= -1.549, p=0.121

IQR: inter quartile range.

In both genders a lower CSI-D COGSCORE was significantly associated with having had no formal education (U=34866.5, z= –6.688, p<0.001, for females; U=20757.0, z= –6.278, p<0.001, for males). After adjusting for the effect of age, having no formal education was significantly associated with greater odds of having 'probable dementia' by CSI-D, see Table 2.

**Table 2:** Logistic regression model of education as a predictor of having ‘probable dementia’ by ‘CSI-D’.

			B	Significance	OR 95% CI for OR	Lower	Upper
Females	No formal education		1.324	< 0.001	3.757	2.207	6.394
	Age band	70-74 years		0.018	1		
		75-79 years	0.312	0.214	1.366	0.835	2.235
		80-84 years	-0.392	0.287	0.676	0.329	1.389
		85 years and over	0.678	0.014	1.971	1.150	3.379
	Constant		-2.654	< 0.001	0.070		
Males	No formal education		1.546	< 0.001	4.693	2.582	8.529
	Age band	70-74 years		0.425	1		
		75-79 years	-0.394	0.270	0.674	0.335	1.357
		80-84 years	-0.738	0.130	0.478	0.184	1.243
		85 years and over	-0.193	0.638	0.825	0.369	1.842
	Constant		-2.640	< 0.001	0.071		

OR: odds ratio; CI: confidence interval.

**Phase II assessments.** In phase II we fully assessed 168 (91.3%) of the people with 'probable dementia', 56 (53.8%) people with 'possible dementia' and 72 (7.9%) people with 'no dementia' according to CSI-D score, giving a stratified sample of 296.

Gender specific regression models were constructed to investigate the influence of schooling on various measures of cognitive function. CSI-D COGSCORE, CERAD 10 word list score and DSM-IV dementia diagnosis were all investigated in univariate and age-adjusted models, see Table 3. Males with no formal education were significantly more likely to perform poorly on the CSI-D COGSCORE and on the CSI-D overall. This effect was not seen in females, and this is likely to be due, in part, to the lower levels of schooling in females limiting statistical power.

**Table 3:** Association between markers for cognitive function and schooling in phase II cohort.

		Univariate odds ratio (95% CI, significance)	Age adjusted odds ratio (95% CI, significance)
Females	CSI-D patient cognitive score	1.01 (0.96 to 1.05, p=0.786)	1.00 (0.95 to 1.04, p=0.858)
	CERAD 10 word list score	1.13 (0.97 to 1.31, p=0.115)	1.08 (0.92 to 1.26, p=0.339)
	Probable dementia by CSI-D	0.83 (0.61 to 1.11, p=0.211)	0.85 (0.63 to 1.16, p=0.316)
	DSM-IV dementia diagnosis	0.91 (0.47 to 1.77, p=0.776)	1.11 (0.55 to 2.22, p=0.774)
Males	CSI-D patient cognitive score	1.11 (1.03 to 1.20, p=0.010)	1.09 (1.01 to 1.18, p=0.028)
	CERAD 10 word list score	1.12 (0.91 to 1.37, p=0.273)	1.09 (0.87 to 1.37, p=0.452)
	Probable dementia by CSI-D	0.48 (0.31 to 0.75, p=0.001)	0.54 (0.34 to 0.86, p=0.010)
	DSM-IV dementia diagnosis	0.44 (0.16 to 1.18, p=0.103)	0.59 (0.20 to 1.76, p=0.345)

Interestingly, performance on the CERAD 10-word learning list and diagnosis of DSM-IV dementia were not associated with having attended school. Performance on the 10-word list was similar in men and women with a median of two words recalled in both groups (U=9142.0, z= –0.603, p=0.547), despite the lower education levels and greater age of the female group, suggesting a lack of educational bias in this assessment.

Of those dementia cases with education data available (n=75), 31 had vascular dementia and 36 had Alzheimer's disease. Rates of education did not vary between these two subtypes, with 10 (32.3%) and 11 (30.6%), respectively, having attended school, odds ratio 1.08 (95% CI 0.38 to 3.04).

**Other markers for cognitive reserve.** Other potential markers of cognitive reserve were also assessed including highest occupation and markers of childhood disadvantage such as head circumference and leg length. There was no association between highest occupation and DSM-IV dementia, although overall 88.6% of those with dementia and 93.7% of those without dementia were agricultural workers, with only 5.8% of those with dementia and 2.5% of those without dementia having previously had a clerical, administrative or professional occupation. Head circumference and leg length did not differ between those with and without DSM-IV dementia for either gender.

## DISCUSSION

This population of older adults in rural Tanzania had a strikingly low overall level of education, particularly so in women. Illiteracy and low educational level were both associated with 'probable dementia' by CSI-D but not with dementia by DSM-IV criteria following a detailed clinical interview and further cognitive assessment. We will now consider possible reasons for this finding.

**Cognitive assessment by CSI-D.** The CSI-D was developed and validated in low literacy settings,^42^ more recently forming part of the 10/66 international dementia research group protocol used across LMIC.^35^

In other studies of dementia in SSA, the CSI-D has been used alone to assess for dementia,^28^ cognitive impairment^4^ and as a screening tool for dementia.^29^ CSI-D COGSCORE totals of 28/33^28^ and 25.5/33^4^ have been used as indicative of cognitive impairment or dementia respectively in SSA studies of dementia prevalence. In Hai, overall cognitive performance on the CSI-D COGSCORE was lower than might be expected, with median COGSCORE totals of only 25.7 for women and 27.7 for men.

In the original validation of the CSI-D across high and low literacy settings, mean COGSCORES for those who did not have dementia ranged from 28-30 with the exception of a cohort in Nigeria who had similar educational levels to our cohort in Hai and mean COGSCORES of 25.42

Although the 10/66 protocol has been developed for use in LMIC countries, those with increased educational level, particularly secondary education and higher, are recognised to perform better that those without education on cognitive testing using this protocol.^10^ In Hai, the overall level of literacy and school attendance was low in comparison with sites such as India and China, included in the published study. Normative data have not been published for the SSA 10/66 study cohort, possibly due to the small numbers involved, so direct comparisons are difficult, but low observed CSI-D scores might be due to differences in population norms for performance in a very low literacy setting.

It is recognised that even one year of school attendance can markedly influence performance on cognitive testing, not only in tasks dependent on ability to read or write but in other tests of cognitive ability.^43^ In Hai, we found an independent association between school attendance and CSI-D cognitive score in in men, but not in women, possibly due to the low numbers of women who had attended formal education.

The CSI-D also includes a relative interview (RELSCORE) which is given proportionally more weight in scoring when compared to the cognitive section. Differing baseline cognitive ability cannot therefore be the only factor affecting CSI-D dementia. Nevertheless, those without formal education were more likely to have 'probable dementia' by CSI-D indicating that they had some degree of cognitive impairment, which was apparent to their relatives.

**Other cognitive assessments.** As part of the 10/66 research group protocol, all participants seen in the second phase of the study were assessed using the CERAD 10 word delayed recall learning list.^40^ This list has been modified slightly from its original form prior to inclusion in the 10/66 protocol, and involves reading a list of 10 familiar words to the participant before asking them to immediately recall words from the list. The list is presented three times in a similar manner, and participants are then asked to recall these words after an interval of several minutes. This word list used alone detected dementia with a specificity and sensitivity of 73.5% and 76.9% respectively in the Ibadan-Indianapolis study.^30^ Performance on this task has been reported to differ according to literacy status.^43^ However, population norms of performance on this test across LMIC indicate that although variations in performance exist, delayed recall performance for cognitively intact elderly is fairly consistent.^44^ In Hai, we found that in contrast to the CSI-D, performance on the 10 word learning list did not appear to be influenced by educational level or literacy with a median score on delayed recall of 2/10. Performance was notably lower than that reported in other LMIC countries,^44^ but this task did appear to be less educationally biased than other cognitive assessment tools in our cohort.

**Comparison with other studies conducted in SSA.** In contrast to the majority of studies from high-income countries, those conducted in SSA have, for the most part, not found an association between dementia on clinical assessment and years of education.^29,30^ Although the overall number of conducted studies are few, educational level is broadly similar to that reported in Hai, with illiteracy levels ranging between 47%^31^ and 97%.^4^ Those studies which do report an association describe populations markedly different to that in Hai. In an urban, mostly male cohort in Senegal, a significant association between illiteracy and dementia was reported.^33^ Another study from Nigeria found that at least some primary education was protective for dementia,^28^ however this study used the CSI-D only when making dementia diagnosis. The proportion of participants having no formal education was 76%, higher than found in Hai and 89% of dementia cases were female. It may therefore be the case that those performing badly on the CSI-D were also those with lower levels of formal education, similarly to those found in our cohort in Hai. In general, in SSA, cognitive impairment assessed using screening tools, rather than clinical diagnosis, is associated with lower levels of formal education.^45^ Prevalence of cognitive impairment also appears relatively high^45,46^ when such screening tools are used to provide a diagnosis. In contrast, those studies including a clinical assessment for dementia report a lack of association with educational level and a lower prevalence of dementia than cognitive impairment. Overall in SSA, educational level appears to be associated with lower cognitive performance, despite use of tools adapted to low-literacy settings. Since this lower cognitive performance is not generally associated with higher levels of dementia following clinical assessment, education is unlikely to be a robust marker of cognitive reserve in these settings.

**Educational attainment.** Most studies of dementia and educational level have been conducted in settings where the overall level of education is significantly higher than in our cohort. In these settings, years of education are likely to be related to educational attainment, with those continuing with education likely to be more able. Cognitive performance measured at age 11^11^ or in young adults^47^ has been found to correlate with late life cognitive ability independently of neuropathological evidence of dementia. Educational level could in these situations be a valid marker of cognitive reserve. Likewise, failure to complete education in these settings is likely to be a marker of more general social disadvantage which is likely to have an impact in terms of cognitive reserve. In this cohort where access to education appears to have been very limited, years of education are less likely to reflect childhood intelligence.

**Literacy.** Studies of older adults in high-income countries suggest that amongst cohorts with a low educational level, self-reported literacy may be a better measure of educational attainment than years of schooling.^48^ A meta-analysis of illiteracy and dementia risk reported a strong association.^19^ Studies from Nigeria have reported a proportion of individuals learning to read and write through informal methods such as attendance at religious education classes.^29,31^ In Hai, few participants reported having attended adult education classes, and only 1% of those who had no formal schooling were able to read and write, suggesting that in Hai informal or alternative education was not prevalent. Literacy is therefore not necessarily a more robust measure of childhood intelligence or cognitive reserve in this setting.

**Education and social disadvantage.** In high-income settings, low educational level is often associated with other markers of childhood social disadvantage, also affecting cognitive reserve. The Ibadan-Indianapolis study, which compared African Americans living in Indianapolis with Yoruba living in Nigeria, found that low educational attainment was associated with dementia in the American cohort but not in the Yoruba. In the Indianapolis cohort low educational level was highly correlated with rural residence and occupation. The prevalence of dementia was also significantly lower in the Yoruba despite a comparatively low level of educational attainment.

In the Ibadan Study of Ageing, in Nigeria, there was a linear relationship between socioeconomic status, rural residence and dementia.^30^ Educational level did not show this association. Educational level may therefore be less associated with other markers of disadvantage in this setting.

Since so many women had no schooling it is likely that failure to attend school was in part a cultural norm and not the marker of deprivation that it might be in high income countries. During the course of our research it became clear that many older women had married and started a family at ages where they might have been expected to be at school in high-income countries.

**Occupation and rural residence.** Occupational level has been suggested as an alternative potential measure of cognitive reserve due to difficulties in comparing studies of educational level.^9^ Almost 90% of our participants were agricultural workers and we did not find any association between dementia and occupational attainment, possibly due to the small number of individuals recording other occupations. We found no association between rural residence and dementia risk, again maybe due to the vast majority reporting lifetime rural residence.

**Limitations.** We relied on self and family report of literacy and educational level and made no attempt to assess this formally. In addition, the phase II cohort was stratified, with over-sampling for poor performance on the CSI-D. Although this may have resulted in some bias, the themes explored in this study are unlikely to have been significantly affected by this.

**Conclusion.** As reported from other studies in SSA, we did not find an independent association between dementia on DSM-IV criteria and educational level. The reasons for this are unclear. One possibility is that in this rural cohort, most of whom reported having never lived outside the area and having only worked in agriculture, low educational level is not the marker of social and economic disadvantage that it might be in other settings where access to education is more widespread. Years of education might also not be a good marker of actual educational attainment, or indeed cognitive reserve, in this setting for the same reason. Performance on a screening tool designed for use in developing countries also appeared markedly lower than in other settings even in SSA. Similar findings are reported in other SSA studies where generally cognitive impairment on formal testing appears to be related to years of education, but prevalence of dementia is much lower when diagnosed on clinical assessment and generally not related to educational level. Alternative methods of measuring cognitive reserve in SSA and similar settings are therefore likely to be needed for identification of modifiable risk factors for dementia.

Despite the existence of assessment tools for dementia designed for developing country settings, these do appear to remain educationally biased when used in settings such as Hai with a very low level of access to education, and high rate of illiteracy. This results in difficulties in assessing the true relationship between dementia and education in these settings.

Worldwide, one in five adults is reported to be illiterate with two thirds of these being women (UNESCO).^49^ In Tanzania, although we reported a high level of illiteracy in this elderly cohort, current estimates suggest a 98% rate of primary school enrolment for current school aged children. A 73% adult literacy rate was reported in 2011. The demographic profile of the current population of Tanzania may have changed considerably. It remains to be seen how this might affect dementia risk in future.

## References

[1] Prince M, Guerchet MMP (2013). Policy Brief for Heads of Government: The Global Impact of Dementia
2013-2050.

[2] George-Carey R, Adeloye D, Chan KY (2012). An estimate of the prevalence of dementia in Africa: A systematic
analysis. J Glob Health.

[3] Longdon AR, Paddick SM, Kisoli A (2013). The prevalence of dementia in rural Tanzania: a cross-sectional
community-based study. Int J Geriatr Psychiatry.

[4] Guerchet M, Houinato D, Paraiso MN (2009). Cognitive impairment and dementia in elderly people living in
rural Benin, west Africa. Dement Geriatr Cogn Disord.

[5] Guerchet M, M'Belesso P, Mouanga AM (2010). Prevalence of dementia in elderly living in two cities of Central
Africa: the EDAC survey. Dement Geriatr Cogn Disord.

[6] Paraïso MN, Guerchet M, Saizonou J (2011). Prevalence of Dementia among Elderly People Living in Cotonou, an
Urban Area of Benin (West Africa). Neuroepidemiology.

[7] Whalley LJ, Deary IJ, Appleton CL, Starr JM (2004). Cognitive reserve and the neurobiology of cognitive
aging. Ageing Res Rev.

[8] Fratiglioni L, Wang HX (2007). Brain reserve hypothesis in dementia. J Alzheimers Dis.

[9] Valenzuela MJ, Sachdev P (2006). Brain reserve and dementia: a systematic review. Psychol Med.

[10] Prince M, Acosta D, Ferri CP (2012). Dementia incidence and mortality in middle-income countries, and
associations with indicators of cognitive reserve: A 10/66 Dementia Research
Group population-based cohort study. Lancet.

[11] Murray AD, Staff RT, McNeil CJ (2011). The balance between cognitive reserve and brain imaging
biomarkers of cerebrovascular and Alzheimer's diseases. Brain.

[12] Mortimer JA, Snowdon DA, Markesbery WR (2003). Head circumference, education and risk of dementia: findings from
the Nun Study. J Clin Exp Neuropsychol.

[13] Meng X, D'Arcy C (2012). Education and dementia in the context of the cognitive reserve
hypothesis: a systematic review with meta-analyses and qualitative
analyses. PLoS ONE.

[14] Letenneur L, Launer LJ, Andersen K (2000). Education and the risk for Alzheimer's disease: sex makes a
difference. EURODEM pooled analyses. EURODEM Incidence Research
Group. Am J Epidemiol.

[15] Cobb JL, Wolf PA, Au R, White R, D'Agostino RB (1995). The effect of education on the incidence of dementia and
Alzheimer's disease in the Framingham Study. Neurology.

[16] Lindsay J, Laurin D, Verreault R (2002). Risk Factors for Alzheimer's Disease: A Prospective Analysis from
the Canadian Study of Health and Aging. Am J Epidemiol.

[17] Stern Y, Gurland B, Tatemichi TK, Tang MX, Wilder D, Mayeux R (1994). Influence of education and occupation on the incidence of
Alzheimer's disease. JAMA.

[18] Caamano-Isorna F, Corral M, Montes-Martinez A, Takkouche B (2006). Education and dementia: a meta-analytic study. Neuroepidemiology.

[19] Brucki SMD (2010). Illiteracy and dementia. Dement Neuropsychol.

[20] Sharp ES, Gatz M (2011). Relationship between education and dementia: an updated
systematic review. Alzheimer Dis Assoc Disord.

[21] Dong MJ, Peng B, Lin XT, Zhao J, Zhou YR, Wang RH (2007). The prevalence of dementia in the People's Republic of China: a
systematic analysis of 1980-2004 studies. Age Ageing.

[22] Zhou DF, Wu CS, Qi H (2006). Prevalence of dementia in rural China: impact of age, gender and
education. Acta Neurol Scand.

[23] Zhang MY, Katzman R, Salmon D (1990). The prevalence of dementia and Alzheimer's disease in Shanghai,
China: impact of age, gender, and education. An Neurol.

[24] Farrag A, Farwiz HM, Khedr EH, Mahfouz RM, Omran SM (1998). Prevalence of Alzheimer's disease and other dementing disorders:
Assiut-Upper Egypt study. Dement Geriatr Cogn Disord.

[25] Afgin AE, Massarwa M, Schechtman E (2012). High prevalence of mild cognitive impairment and Alzheimer's
disease in arabic villages in northern Israel: impact of gender and
education. J Alzheimers Dis.

[26] Chandra V, Ganguli M, Pandav R, Johnston J, Belle S, DeKosky S (1998). Prevalence of Alzheimer's disease and other dementias in rural
India. The Indo-US study. Neurology.

[27] Shaji S, Bose S, Verghese A (2005). Prevalence of dementia in an urban population in Kerala,
India. Brit J Psychiatry.

[28] Ochayi B, Thacher TD (2006). Risk factors for dementia in central Nigeria. Aging Ment Health.

[29] Ogunniyi A, Baiyewu O, Gureje O (2000). Epidemiology of dementia in Nigeria: results from the
Indianapolis-Ibadan study. Eur J Neurol.

[30] Gureje O, Ogunniyi A, Kola L (2006). The profile and impact of probable dementia in a sub-Saharan
African community: results from the Ibadan Study of Aging. J Psychos Res.

[31] Yusuf AJ, Baiyewu O, Sheikh TL, Shehu AU (2011). Prevalence of dementia and dementia subtypes among
community-dwelling elderly people in northern Nigeria. Int Psychogeriatr.

[32] Guerchet M, Mouanga AM, M'Belessoa P (2012). Factors associated with dementia among elderly people living in
two cities in central Africa: The EDAC multicenter study. J Alzheimers Dis.

[33] Coume M, Toure K, Thiam MH (2012). Estimate of the prevalence of cognitive impairment in an elderly
population of the health center of Senegalese national retirement
institution. GeriatrEt Psychol Neuropsychiatr Vieil.

[34] Chandra V, Ganguli M, Ratcliff G (1998). Practical issues in cognitive screening of elderly illiterate
populations in developing countries. The Indo-US Cross-National Dementia
Epidemiology Study. Aging (Milano).

[35] Prince M, Acosta D, Chiu H, Scazufca M, Varghese M (2003). Dementia diagnosis in developing countries: a cross-cultural
validation study. Lancet.

[36] Hall KS, Hendrie HC, Brittain HM (1993). The Development of a Dementia Screening Interview in 2 Distinct
Languages. Int J Meth Psychiatr Res.

[37] Chen CH, Mizuno T, Elston R (2010). A comparative study to screen dementia and APOE genotypes in an
ageing East African population. Neurobiol Aging.

[38] Copeland J, Dewey M, Griffith-Jones H (1986). A computerised psychiatric diagnostic system and case
nomenclature for elderly subjects: GMS and AGECAT. Psychol Med.

[39] Cummings JL, Mega M, Gray K, Rosenberg-Thompson S, Carusi DA, Gornbein J (1994). The Neuropsychiatric Inventory: comprehensive assessment of
psychopathology in dementia. Neurology.

[40] Welsh KA, Butters N, Mohs RC (1994). The Consortium to Establish a Registry for Alzheimer's Disease
(CERAD). Part V. A normative study of the neuropsychological
battery. Neurology.

[41] American Psychiatric Association (1994). Diagnostic and statistical manual of mental disorders.

[42] Hall KS, Gao S, Emsley CL, Ogunniyi AO, Morgan O, Hendrie HC (2000). Community screening interview for dementia (CSI 'D'); performance
in five disparate study sites. Int J Geriatr Psychiatry.

[43] Nitrini R, Caramelli P, Herrera E Jr (2004). Performance of illiterate and literate nondemented elderly
subjects in two tests of long-term memory. J Int Neuropsychol Soc.

[44] Sosa AL, Albanese E, Prince M (2009). Population normative data for the 10/66 Dementia Research Group
cognitive test battery from Latin America, India and China: a
cross-sectional survey. BMC Neurology.

[45] Onadja Y, Atchessi N, Soura BA, Rossier C, Zunzunegui M-V (2013). Gender differences in cognitive impairment and mobility
disability in old age: A cross-sectional study in Ouagadougou, Burkina
Faso. Arch Gerontol Geriatr.

[46] Clausen T, Romoren TI, Ferreira M, Kristensen P, Ingstad B, Holmboe-Ottesen G (2005). Chronic diseases and health inequalities in older persons in
Botswana (southern Africa): a national survey. J Nutr Health Aging.

[47] Riley KP, Snowdon DA, Desrosiers MF, Markesbery WR (2005). Early life linguistic ability, late life cognitive function, and
neuropathology: findings from the Nun Study. Neurobiol Aging.

[48] Manly JJ, Schupf N, Tang M-X, Stern Y (2005). Cognitive Decline and Literacy Among Ethnically Diverse
Elders. J Geriatr Psychiatry Neurol.

[49] UNESCO (2014). International Literacy Data Quebec.

